# Use of the interRAI PEDS HC in children receiving home care in Ontario, Canada

**DOI:** 10.1186/s12913-022-08442-z

**Published:** 2022-08-18

**Authors:** Emily Thorburn-Winsor, Megan Doherty, Aaron Jones, Christina Vadeboncoeur

**Affiliations:** 1 Michael G. DeGroote School of Medicine, McMaster University, ON Hamilton, Canada; 2 Children’s Hospital of Eastern Ontario, 401 Smyth Rd, Ottawa, ON K1H 8L1 Canada; 3 University of Ottawa, Ottawa, Ontario Canada; 4 Roger Neilson House, Ottawa, Ontario Canada; 5 Department of Health Research Methods, Evidence, and Impact, McMaster University, Hamilton, ON Canada

**Keywords:** Pediatric home care, Children with medical complexity, Standardised assessment, PEDS-HC, interRAI

## Abstract

**Background:**

There is no standard assessment tool for pediatric home care recipients in Canada, limiting the availability of comparable, population-based data. The objective of this study was to describe pediatric home care recipients who were part of a pilot implementation of the interRAI Pediatric Home Care Assessment Form (PEDS-HC) among medically complex children referred to home care agencies in three regions in Ontario, Canada.

**Methods:**

All 14 agencies providing home care to children in Ontario were invited to participate in the pilot project, and 9 participated in an education session. Three of these agencies used the PEDS-HC during the pilot implementation between February 2018 and March 2020. We used de-identified data to describe the demographics, home care needs, and diagnoses of pediatric home care recipients.

**Results:**

The sample of 474 assessments was predominantly male (60.34%), with an average age at assessment of 12.36 years (SD 4.56). Most (78.48%) reported English as their primary language. Most children assessed had between two and eight medical diagnoses. Diagnoses reported varied: gastrointestinal, musculoskeletal, respiratory and neurological conditions were most common. The prevalence of urinary incontinence (40.1%) and bowel incontinence (70.9%) were high. Over 60% of children were rarely or only sometimes understood. A majority of children had adequate hearing (83.5%) and vision (68.6%). Extensive services were being provided in 10% of children assessed. Most children received care both at school and at home (70.89%), with 20.89% receiving home care only.

**Conclusions:**

The PEDS-HC provides a detailed, standardised descriptive profile of medically complex children receiving home care. Expanding use of PEDS-HC would promote consistency in care planning and delivery on the patient level, enable cross-jurisdictional comparisons, and inform utilization tracking and health care funding decisions on the organization and provincial levels.

**Supplementary Information:**

The online version contains supplementary material available at 10.1186/s12913-022-08442-z.

## Background

The age-adjusted rate of medical complexity for children and youth in Canada in 2015–2016 was 948 per 100,000 children and youth, with wide variation in rates across provinces and territories [1]. Supporting a child to remain at home with their family is crucial to ensuring their quality of life [2]. The care needs of each child are unique, change over time, and often persist throughout their life [3].

The variability in the home and community health care services available to children, youth and families across the province of Ontario is significant [4]. For example, children and youth living in the Ottawa Sub region of the Champlain region of Ontario were shown to have better access to physician services and developmental and rehabilitation services than children and youth living elsewhere in the region [4]. Pediatric home care assessments in Ontario are not standardized, and a variety of tools are being used by different service providers. Because each agency uses different tools, some of which are locally developed, the item content of these tools is not known. Many of these have not been tested for reliability and validity [5]. This may lead to high variation in the services which similar children receive, depending on where and how they are assessed. The care provided is based on a combination of the judgement of the care coordinator and the resources available in a particular region [4]. A coordinated system of care that provides a standardized assessment which is available to all caregivers will maximize the child’s time at home, inform new caregivers of their needs and ensure smooth transitions between settings of care.

Home care services in Ontario can include personal support, home support, nursing support and allied service support including physiotherapy, occupational therapy, speech-language pathology, dietetics and social work. Pediatric nursing support is considered a specialized type of nursing support. Advances in pediatric health care in recent decades have meant that children now survive conditions that were untreatable in the past. Hospital based interventions including tracheostomy and long term ventilation, long term intravenous feeding, and insertion of feeding tubes, among other advances, have allowed children to survive with increasing reliance on technology and many hours of hands on care per day. Home and community based medical care has not yet matched the needs of these children when they go home from hospital. Parents are expected to manage complicated medical care for their children at home alone, struggling to stay employed and healthy themselves [6].

The interRAI Pediatric Home Care Assessment Form (PEDS-HC) is a standardized assessment tool specifically designed to assess the long-term community-based service and support needs of children and youth (aged 4–20 years) with a wide range of chronic physical or behavioral health challenges [7]. The 20 section form consists of questions regarding demographic data, services currently provided and utilised, cognitive abilities, communication and vision, mood and behaviour, psychosocial wellbeing, functional status, continence, medical history and diagnoses, medications used, treatments and procedures, social supports, and environmental assessment. The RAI Home Care Assessment form was used as the core from which to develop the PEDS-HC to allow the development of a “picture” of home care users at an early age that could be integrated into a tool already in use in an adult population [6]. Changes from the adult instrument included demographic changes related to describing the setting where the child lives, presence or absence of intellectual or developmental disabilities in the child, and mental health problems in either the child or their caregivers (or both) [6]. Initial research in the United States indicates that PEDS-HC items exhibit good predictive validity, and the tool has been implemented to assess children in Medicaid programs in 2 US states (New York and Maryland), providing seamless integration with these states’ use of the RAI adult home care assessments. The PEDS-HC has not yet been implemented in a Canadian context. The suite of interRAI instruments are a global standard, supported by research developed by over 100 physicians and researchers across 35 countries [8]. A number of these instruments including the interRAI Home Care assessment tool are used in Ontario in a variety of care settings, including adult home care, acute care, palliative care and mental health [8–10]. Data collected from these assessments is being used by agencies such as the Canadian Institute for Health Information to inform policy on provision of care in Canada [11] – unfortunately the information currently collected includes only those over the age of 18.

We implemented the PEDS-HC on a pilot basis in three regions of Ontario, to comprehensively assess the service and support needs of children and youth (aged 4–18 years – the age range for children used for funding in Ontario)) receiving pediatric home-care services for more than 6 weeks. The objective of this study is to describe the pediatric home care recipients who were assessed during the pilot implementation of the PEDS-HC in Ontario.

## Methods

### Study design and setting

This is a cross-sectional pilot study of pediatric home care clients in Ontario, Canada. PEDS-HC assessments were conducted between February 1, 2018 and March 31, 2020 in three health regions in Ontario, Canada (Central, Central East, and Hamilton-Niagara-Haldimand-Brant).

### Data sources

All data was extracted from the pilot PEDS-HC implementation. Extracted data included demographics of participants, level of care required (e.g. incontinence of bowel/bladder, cognition, communication abilities), diagnoses and services utilised. The PEDS-HC was developed for children with complex medical needs in Texas, USA [12]. Research indicates that the PEDS-HC demonstrates good predictive validity [7]. The assessment was administered by trained home care coordinators.

### Participants

Participants in this study represent a convenience sample of pediatric home care recipients in the three pilot regions. The Central region completed assessment on 49 children, the Central East region completed assessments on 52 children, and the Hamilton Niagara Haldimand Brand region completed assessments on 373 children. Children were included in the study if they were between 4 and 18 years of age and were expected to require pediatric home-care services for at least 3 months. Children were excluded at the discretion of the care coordinator, and care coordinators did not include children for whom the duration of homecare was expected to be short or if the parent appeared unduly stressed by their child’s clinical condition.

### Measures

Primary measures of interest included demographic characteristics (age, gender, language), service use (care delivered at school, at home or both), clinical characteristics (expression/comprehension, vision/hearing, bladder/bowel incontinence) and diagnoses. Age at assessment was calculated based on date of birth reported and date of assessment.

### Analysis

Summary statistics of the demographic and clinical characteristics of participants were reported. As many clients had several diagnoses, we grouped diagnoses by category and reported frequencies per category visualised by a histogram. To further illustrate the types of conditions for participants with multiple diagnoses, we constructed a Venn diagram of participants who had musculoskeletal, respiratory diagnoses, or neurological diagnoses.

All methods were carried out in accordance with relevant guidelines and regulations stipulated by the Province of Ontario Ministry of Health and by the Canadian Good Clinical Practices and Canadian Biomedical Ethics Research Guidelines.

## Results

### Demographic and clinical characteristics of children receiving community care

There were 474 children who were assessed using the PEDS-HC during this study. Sample characteristics along with means and percentage of population are presented in Table 1. The majority of study participants were male (60.3%). The average age at assessment of 12.4 years (SD 4.56). Most children (78.5%) had a primary language of English. The prevalence of urinary incontinence (40.1%) and bowel incontinence (70.9%) were high. Over 60% of children were rarely or only sometimes understood. A majority of children had adequate hearing (83.5%) and vision (68.6%). The majority of participants received both school and home-based community care (70.9%), with 20.9% receiving only home care.

**Table 1:** Demographic and clinical characteristics of children receiving home care (*n* = 474, except where otherwise noted)

Variable	Value (percentage)
Gender	
Male	286 (60.3%)
Female	188 (39.6%)
Age at Assessment (*n* = 472, 99.6%)	
4–6	64 (13.6%)
7–10	102 (21.6%)
11–14	128 (27.1%)
15–18	132 (28.0%)
18+	46 (9.7%)
Mean age (SD)	12.4 (4.6)
Primary Language	
English	372 (78.5%)
Arabic	18 (3.8%)
Missing	63 (13.3%)
Other	21 (4.4%)
Additional Supports received^a^	
No support	20 (4.2%)
Home support	99 (20.9%)
School support	19 (4.0%)
Both Home and School	336 (70.9%)
Child’s Ability to Express Him/Herself	
Understood	91 (19.1%)
Usually understood	37 (7.8%)
Often understood	59 (12.4%)
Sometimes understood	141 (29.7%)
Rarely or never understood	146 (30.8%)
Child’s Comprehension Ability	
Understands	113 (23.8%)
Usually Understands	58 (12.2%)
Often Understands	57 (12.0%)
Sometimes understands	155 (32.7%)
Rarely or never understands	91 (19.2%)
Hearing	
Adequate	396 (83.5%)
Minimal Difficulty	31 (6.5%)
Moderate Difficulty	18 (3.8%)
Severe difficulty	26 (5.5%)
No Hearing	3 (0.6%)
Vision	
Adequate	325 (68.6%)
Minimal Difficulty	71 (15.0%)
Moderate Difficulty	31 (6.5%)
Severe Difficulty	37 (7.8%)
No Vision	10 (2.1%)
Communication Aide	
Yes	178 (37.5%)
No	296 (62.4%)
Hearing Aid	
Yes	38 (8.0%)
No	436 (92.0%)
Visual aid Including Glasses	
Yes	107 (22.6%)
No	367 (77.4%)
Urinary Incontinence	
Yes	284 (40.1%)
No	190 (59.9%)
Bowel incontinence	
Yes	336 (70.9%)
No	138 (29.1%)
Urinary Collection Device	
None	465 (98.1%)
Condom catheter	0 (0.0%)
Indwelling catheter	4 (0.8%)
Cystostomy, nephrostomy, or ureterostomy	5 (1.0%)
Bowel Collection Device	
Colostomy	6 (1.3%)
Ileostomy	1 (0.2%)
None	467 (98.5%)

^a^ Additional supports received includes support workers, nursing, physiotherapy, occupational therapy and speech language pathology, mobility, behavioural interventions, etc.

**Fig. 1:**
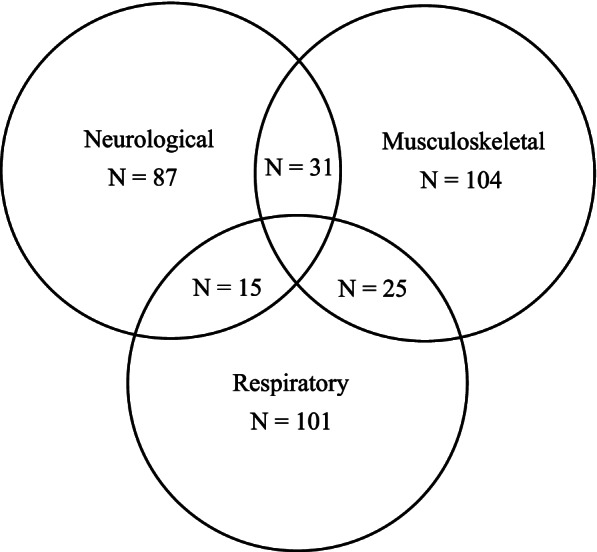
Histogram of number of diagnoses reported in sample population

Frequencies of disease categories are presented in Table 2. The most common diagnostic categories were mental health and developmental disorders. Figure 1 shows a histogram of the number of diagnoses reported for each child. The median number of diagnoses for children was 4, with a 25th percentile of 3 and 75th percentile of 6.

**Table 2:** Disease categories

Category (*n* = 474)	Number with diagnosis (%)
Mental health and developmental	399 (84.2%)
Gastrointestinal	135 (28.5%)
Musculoskeletal	104 (21.9%)
Respiratory	101 (21.3%)
Neurological	87 (18.4%)
Cardiovascular	47 (9.9%)
Metabolic disorders	41 (8.7%)
Infections	37 (7.8%)
Other *	265 (55.9%)

* includes blindness (*n* = 49), cancer (*n* = 7), cleft palate (*n* = 6), deafness (*n* = 48), explicit terminal prognosis (*n* = 8), failure to thrive (*n* = 14), renal failure (*n* = 8), and other (*n* = 125)

**Fig. 2:**
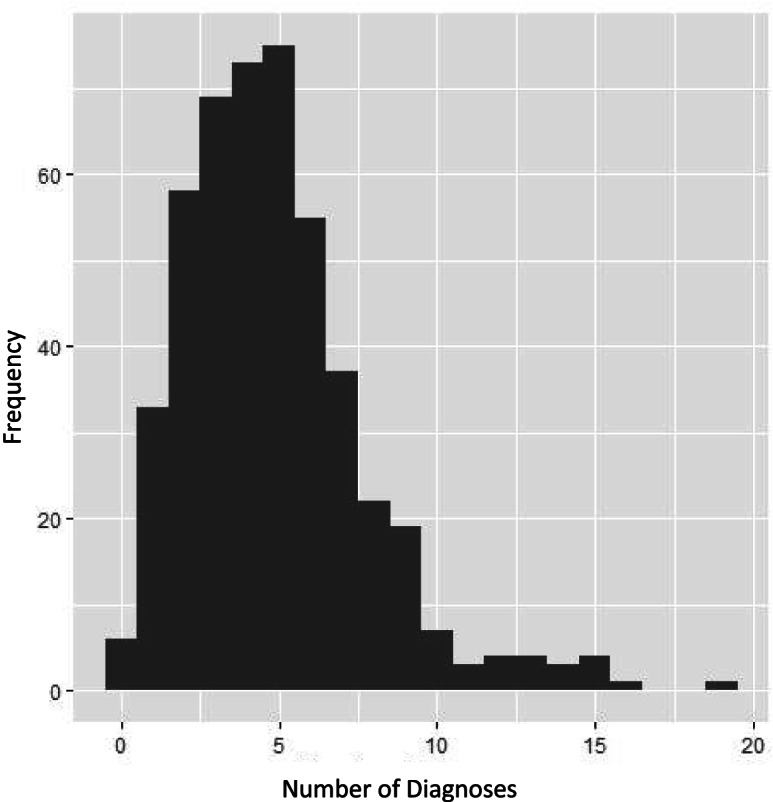
Venn Diagram showing the overlap of diagnostic categories in the sample population

There was significant diagnostic overlap in children who had neurological, respiratory and/or musculoskeletal diagnoses (Fig. 2). There were 25 children (5.3%) with diagnoses in all three of these diagnostic categories. These children had high prevalence rates of blindness and deafness (see Supplementary Table 1).

Communication disorders (53.0%), intellectual disability (47.3%), learning disorder (29.3%), motor disorders (28.9%) and autism spectrum disorder (25.7%) were most prevalent mental health or developmental issues reported in the sample population (Table 3).

**Table 3:** Mental health or developmental issues

Diagnosis (*n* = 474 unless otherwise noted)	Number of participants with diagnosis n (%)
Communication disorder	251 (52.9%)
Intellectual disability	224 (47.3%)
Epilepsy/seizure disorder	176 (37.1%)
Learning disorder	139 (29.3%)
Motor disorder	137 (28.9%)
Autism spectrum disorder	122 (25.7%)
Cerebral Palsy	109 (23.0%)
Feeding and eating disorders	74 (15.6%)
Attention deficit hyperactivity disorder (ADHD)	48 (10.1%)
Sleep wake disorders	45 (9.5%)
Anxiety disorder	40 (8.4%)
Obsessive compulsive and related disorders	22 (4.6%)
Oppositional, impulsive-control and conduct disorders	18 (3.8%)
Trauma and stress-related disorder	10 (2.1%)
Depressive disorders	8 (1.7%)
Substance related and addictive disorders	3 (0.6%)
Schizophrenia and other psychotic disorders	2 (0.4%)
Bipolar and related disorders	1 (0.2%)

An analysis of case-mix using the model developed by Phillips [13] divided children into five main groups, with children reported in the group which matched their highest care needs. For example, children classified as needing extensive services may also have impaired cognition and/or reduced physical functioning but were classified as needing extensive services, but children who did not need the components listed below of extensive services, special care or complex care but who had impaired cognition were classified into the impaired cognition group. In 11% of children assessed extensive services were required, including one or more of IV feeding, suctioning, tracheostomy care, oxygen, ventilator or a diagnosis of coma. Special care is defined as needing at least moderate help with selected activities of daily living plus at least one of: a diagnosis of Cystic Fibrosis, IV medication, hospice (receipt of palliative care services in the past 3 years), shift nursing, hospital admission in the past 3 days, or uncontrolled seizure disorder was present in 25% of children. Complex care was defined with the need for at least moderate help with selected activities of daily living plus at least on of: cerebral palsy, explicit terminal prognosis, contractures, hydro/microcephaly, bed or chair bound, pressure ulcer or skin lesion, recurrent aspiration or any plegia and was seen in 13% of the children assessed. Children with impaired cognition made up 17% of the children assessed. Reduced physical functioning was seen in 34% of children assessed.

## Discussion

An essential step to providing equitable health care resources to children at home and at school is the standardized collection of demographic, clinical, and home care need-related data. This type of standardised data is not currently collected on pediatric home care recipients in Canada. The pilot implementation of the PEDS-HC generated a descriptive profile of medically complex pediatric home care recipients in Ontario and demonstrates the range of data available from the assessment. A qualitative study of homecare coordinators involved in this pilot study of the PEDS-HC demonstrated that a standardized list of items to assess was useful in identifying the care needs of children in the home in order to avoid acute care utilization, such as hospitalization [14].

The PEDS-HC was developed in Texas to assess children and youth facing special healthcare challenges to determine their need for long-term community-based living services and support, and demonstrated good predictive validity in a US cohort. It has been implemented to assess the home care needs of children in Medicaid programs in the states of New York and Maryland, where its adoption has the advantage of a seamless transition to the corresponding adult home care tool, the RAI-HC. The RAI-HC has been used to collect standardized data for adults receiving home care in eight Canadian provinces and more than 10 countries internationally including the United States, France and New Zealand. The data quality of those assessments has been demonstrated to be useful to inform decision making at the organizational or policy level [15].

The children in this study, although being cared for at home, show similarities with the children in the Texas cohort [7]. In our study the incidence of intellectual disability and cerebral palsy were very similar those reported in the cohort from Texas (47.3% vs. 46.6 and 23.0% vs 23.4% respectively). The incidence of epilepsy was 37% compared to 29% in Texas. Our cohort had a higher incidence of Autism Spectrum Disorder (26%), compared to 17% in the Texas cohort. Conversely, the incidence of ADHD was lower in our participants (10%) compared to 25% in Texas. This type of comparison of medically complex children across varying jurisdictions is only possible given the implementation of standardised assessments such as the PEDS-HC.

Nearly half (49%) of the children in this study fit into categories of needing extensive services, special care or complex care. The remaining children had either impaired cognition or reduced physical functioning. As a result, 71% of these children received additional medical care supports both at home and at school, and 25% received supports either at home or at school. The categorizations as outlined are dependent on a subset of activities of daily living (eating, bed mobility, toileting and ability to transfer). Although the children range in age from 4 to 18 (20 in the Texas cohort), typical children would be independent in all of these activities, so reliance on caregivers to assist or perform these activities adds significantly to the care the child requires.

Care of children with complex medical needs requires a coordinated multi-disciplinary approach, with services provided by multiple health and social service agencies in both community and facility-based settings [16]. Care planning across multiple services and settings require continuity and information sharing. Without coordination, families struggle to access the services their children require and can be overwhelmed by multiple bureaucratic structures [17]. Geographical considerations in Ontario (and in Canada) increase the complexity of service provision. Standardised assessment tools, such as the PEDS-HC, which can be shared between agencies are cost effective and beneficial in standardising the allocation of support for those living with long term medical conditions [18]. Reassessment of the child’s clinical status at regular intervals such as every 6 months would allow identification of any changes in health condition, either improvement which could lead to a decreased need for home care services, or signs of a change which could be proactively addressed to direct investigations or referrals to optimize care. The PEDS-HC could be a beneficial tool to collect standardised data that can be used by home care coordinators and provider agencies and in determining care plans as well as by policy makers in tracking population-level pediatric home care use and making decisions about health care funding. In addition, in regions where adult version of the tool is used (such as Ontario), the PEDS-HC assessment is intentionally built to allow seamless flow of information into the adult assessment tool. The interRAI suite of instruments includes clinical assessment protocols which are person-centered assessment systems that inform and guide comprehensive care and service planning in different settings and programs. At this time, the PEDS-HC has no such clinical assessment protocols, but the value these would have in individual patient care has been recognized by the homecare providers involved in this pilot study [11]. The PEDS-HC is the only comprehensive, validated tool to assess home care needs for children. Data collected with this tool could be used to inform decisions for human health resources in the pediatric population, similarly to how data collected for adults in the corresponding adult home care tool is currently being used for adult populations in Ontario and in other provinces in Canada.

## Limitations

Limitations of this study include the inherent inability of the sample to be generalised to the entire pediatric home care population in Canada. In addition to this, the PEDS-HC has not been validated in a Canadian population, although it has been validated in neighbouring jurisdictions.

This project was undertaken to explore and address inequities in homecare provision which have increased as technological advances in medicine have meant more children are cared for at home with increasing medical technology. The data collection ranged from 2018 to 2020, and thus is unable to describe whether these inequities have changed during pandemic conditions.

## Conclusion

A standardised assessment for pediatric home care recipients in Ontario such as the PEDS-HC would enable collection of data that can reduce variability and promote equity in care services, enable cross-jurisdictional comparisons, and support policy and funding decisions regarding pediatric home care in Canada.

## Supplementary Information

**Additional file 1: Supplementary Table 1.** Overlap of diagnoses.

## Data Availability

The datasets generated and/or analysed during the current study are not publicly available due privacy legislation but may be made available from upon reasonable request to Dr. Christina Vadeboncoeur (vadeboncoeur@cheo.on.ca) by researchers who can meet criteria for access to the confidential data.

## Abbreviations

PEDS HC: Pediatric Home Care Assessment Form
RAI-HC: Resident Assessment Instrument–Home Care
