# Supplementary material for: Use of the interRAI PEDS HC in children receiving home care in Ontario, Canada
Source: BMC Health Serv Res. 2022 Aug 18;22:1057. doi: 10.1186/s12913-022-08442-z (PMC9389723; doi:10.1186/s12913-022-08442-z)
Supplement: Supplementary file 1 — Additional file 1: Supplementary Table 1. Overlap of diagnoses. [file 12913_2022_8442_MOESM1_ESM.docx]

**Supplementary data:**

| Category | Single | Combined* |
| --- | --- | --- |
| Neurological | Total = 87  Blindness = 20  Deafness = 11 | Total = 31 |
|  |  | Total = 15  Blindness = 3  Deafness = 3 |
| Respiratory | Total = 101  Blindness = 12  Deafness = 15 |  |
|  |  | Total = 25  Blindness = 1  Deafness = 4 |
| Musculoskeletal | Total = 104  Blindness = 14  Deafness = 16 |  |
|  |  | Total = 31  Blindness = 8  Deafness = 7 |

Supplementary Table 1: Overlap of diagnoses

*combined refers to those with both diagnoses, i.e. both neurological and respiratory diagnoses
